# Supplementary material for: Clinical Characteristics of Osimertinib Responder in Non-Small Cell Lung Cancer Patients with EGFR-T790M Mutation
Source: Cancers (Basel). 2019 Mar 15;11(3):365. doi: 10.3390/cancers11030365 (PMC6468762; doi:10.3390/cancers11030365)
Supplement: Supplementary file 1 [file cancers-11-00365-s001.pdf]

Supplementary Material: Clinical Characteristics of Osimertinib Responder in Non-Small Cell Lung Cancer Patients with EGFR-T790M Mutation

Akihiro Yoshimura, Tadaaki Yamada, Naoko Okura, Takayuki Takeda, Kazuki Hirose, Yutaka Kubota, Shinsuke Shiotsu, Osamu Hiranuma, Yusuke Chihara, Nobuyo Tamiya, Yoshiko Kaneko, Junji Uchino and Koichi Takayama

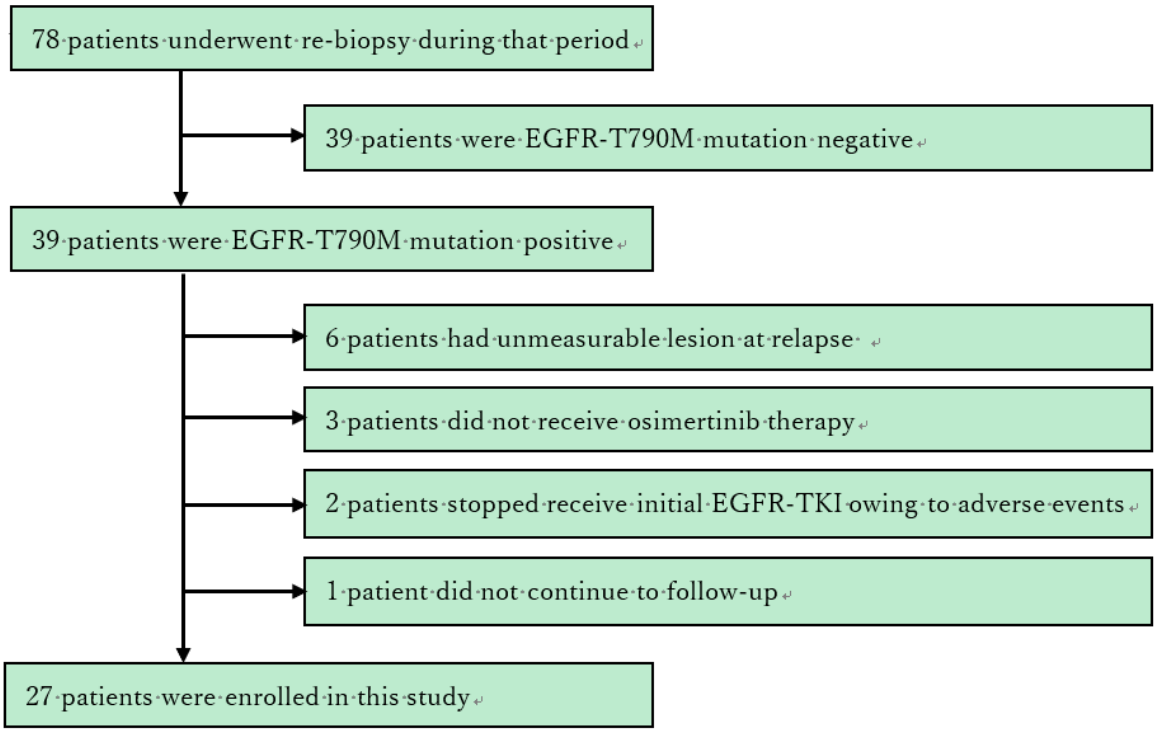

Figure S1. Patient flowchart of NSCLC with EGFR-T790M mutation after they acquired resistance to initial EGFR-TKIs. A total of 78 EGFR-mutant NSCLC patients underwent re-biopsy and 39 NSCLC patients was detected to have the EGFR-T790M mutation. Of these patients, 27 received osimertinib treatment with measurable lesions. In addition, they could continue receiving initial EGFR-TKIs after adverse events until disease progression.

© 2019 by the authors. Licensee MDPI, Basel, Switzerland. This article is an open access article distributed under the terms and conditions of the Creative Commons Attribution (CC BY) license (http://creativecommons.org/licenses/by/4.0/).
